# Occupational Exposure of Plastics Workers to Diisononyl Phthalate (DiNP) and Di(2-propylheptyl) Phthalate (DPHP) in Finland

**DOI:** 10.3390/ijerph17062035

**Published:** 2020-03-19

**Authors:** Simo P. Porras, Minna Hartonen, Jani Koponen, Katriina Ylinen, Kyösti Louhelainen, Jarkko Tornaeus, Hannu Kiviranta, Tiina Santonen

**Affiliations:** 1 Finnish Institute of Occupational Health, PO Box 40, FI-00032 Työterveyslaitos, Finland; minna.hartonen@ttl.fi (M.H.); katriina.ylinen@ttl.fi (K.Y.); klouhelainen@gmail.com (K.L.); jarkkotornaeus@gmail.com (J.T.); tiina.santonen@ttl.fi (T.S.); 2 Finnish Institute for Health and Welfare (THL), PO Box 95, FI-70701 Kuopio, Finland; jani.koponen@thl.fi (J.K.); hannu.kiviranta@thl.fi (H.K.)

**Keywords:** phthalates, occupational exposure, plastic workers, biomonitoring, urine, industrial hygiene, air samples

## Abstract

The aim of this study was to assess occupational exposure to diisononyl phthalate (DiNP) and di(2-propylheptyl) phthalate (DPHP] in Finland. Four companies took part in the research project: A cable factory, a plastic producing company, a producer of coated textiles, and a tarpaulin producer. The cable factory used DPHP (and occasionally also diisodecyl phthalate, DiDP), the plastic producing company used both DPHP and DiNP, and the latter two companies used DiNP in their production. Exposure was assessed by measuring phthalate metabolites in urine samples (biomonitoring) and by performing air measurements. Low-level occupational exposure to DiNP was observed in the company that produced coated textiles—out of eight workers, one extruder operator was exposed to DiNP at levels exceeding the non-occupationally exposed population background levels. Some workers in the cable factory and the plastics producing company were occupationally exposed to DPHP. Air levels of phthalates were generally low, mostly below the limit of quantification. All phthalate metabolite concentrations were, however, well below the calculated biomonitoring equivalents, which suggests that the health risks related to the exposure are low.

## 1. Introduction

Phthalates (phthalate esters) are ubiquitous environmental contaminants that may cause adverse effects on reproduction via their endocrine-disrupting properties. Environmental phthalate exposure has been extensively studied in various countries—even relatively long exposure time-trends are available for many phthalate metabolites in urine samples. Occupational phthalate exposure has been studied much less [1]. This is surprising given that the exposure levels in occupational scenarios may substantially exceed the respective levels in environmental exposure. Some occupational studies do exist—most of them on phthalates, the use of which is currently heavily regulated in Europe (like dibutyl phthalate and di(2-ethylhexyl) phthalate)), and most of these studies have been conducted outside of Europe. Surprisingly, only a few studies exist on occupational exposure to the phthalates still widely used in the industry, such as diisononyl phthalate (DiNP), diisodecyl phthalate (DiDP), and di(2-propylheptyl) phthalate (DPHP) [1].

The data on occupational DiNP exposure consists of five studies [2,3,4,5,6]. In three of these studies, primary metabolite (hydrolytic monoester) monoisononyl phthalate (MiNP) was analyzed. However, today, MiNP is no longer considered a reliable marker of DiNP exposure because it is prone to external DiNP contamination (after abiotic hydrolyzation) and because it is extensively further metabolized into secondary metabolites (oxidized monoesters) of DiNP [2,3,7]. Indeed, the percentage of MiNP in the total amount excreted in urine is low (3%) [8]. Thus, secondary metabolites of DiNP, which are not prone to contamination and are more extensively excreted in urine, are more reliable markers of DiNP exposure.

Two occupational studies exist that have analyzed the secondary metabolite monocarboxy isooctyl phthalate (abbreviated cx-MiOP, cx-MiNP, or MCOP) in post-shift samples of workers. The geometric mean (GM) urinary cx-MiOP concentration of seven workers who used DiNP on polyvinyl chloride (PVC) film manufacturing was 50.5 µg/L (data range 15 to 164 µg/L) [2]. The respective GM of the 11 workers who worked in the same shift but who were not in direct contact with DiNP was 34.1 µg/L (data range 12.2 to 164 µg/L). In both groups of workers, there was a highly significant difference in urinary cx-MiOP resulting from those in the control group (in both cases *p* < 0.0001). Hines et al. also studied the exposure of 12 workers in PVC compounding. Their post-shift urinary results (GM 9.05 µg/L and data range 1.86 to 19.8 µg/L) were statistically different from the mid-shift results of the same workers (*p* = 0.03). These results indicate occupational DiNP exposure of the PVC workers studied.

Koch et al. studied five PVC plastisol workers in Germany [3]. The urinary median cx-MiOP concentration was 57.8 µg/L, and the data ranged from 24.7 to 286 µg/L. Statistical comparison was not made, but concentrations in post-shift samples were approximately 20-times higher than the results of the general population, indicating occupational exposure. Koch and co-workers also analyzed other secondary metabolites of DiNP, namely monohydroxy isononyl phthalate (OH-MiNP) and mono-oxo isononyl phthalate (oxo-MiNP) [3]. The conclusions were the same as with the data on cx-MiOP (see above): Workers were occupationally exposed to DiNP.

Data on occupational DiDP and DPHP exposure are scarce. Koch et al. studied DiDP/DPHP metabolites in the urine of PVC plastisol workers in a car factory in Germany [3]. The workers used a DiNP product that contained small amounts of other phthalates, such as DiDP/DPHP. The urinary concentrations of monohydroxy diisodecyl phthalate (OH-MiDP, median 16.8 µg/L, range 5.6 to 57.6 µg/L), mono-oxo diisodecyl phthalate (oxo-MiDP, median 4.6 µg/L, range 1 to 12.1 µg/L) and monocarboxy isononyl phthalate (abbreviated cx-MiNP, cx-MiDP or MCNP; median 4.7 µg/L, range 2.1 to 20.3 µg/L) were higher than in the respective data on the general population of Germany (median 0.7 µg/L, range <LOQ to 3.4 µg/L; median 0.3 µg/L, range <LOQ to 1.9 µg/L; median 1.0 µg/L, range <LOQ to 5.7 µg/L, respectively). No statistical comparison was made. It should be noted, however, that the analytical separation method used in this work was not able to differentiate the secondary metabolites of DiDP and DPHP, which means that the source of exposure was either DiDP, DPHP, or both.

The aim of this study was to assess phthalate exposure among plastics workers in Finland. Human biomonitoring was the main method for assessing exposure, but we also carried out some air measurements. We compared the urinary levels of workers to the background urinary levels of occupationally non-exposed population [9] and to biomonitoring equivalents (BEs), which were calculated on the basis of available health-based limit values for external exposure.

## 2. Materials and Methods 

### 2.1. Study Population

Potential companies for the study were contacted via telephone or email. Four companies took part in the study: A cable factory (*n* = 5), a plastic producing company (*n* = 5), a producer of coated textiles (*n* = 8), and a tarpaulin producer (*n* = 2). The cable factory used DPHP (CAS 53306-54-0), the plastics producing company used both DPHP (CAS 53306-54-0) and DiNP (CAS 68515-48-0; di-C8-10-branched alkyl esters, C9-rich), and the latter two companies both used DiNP (CAS 28553-12-0) in their production. Diisodecyl phthalate (DiDP, CAS 68515-49-1) was not used during the sample collection campaign, and other phthalates were not used because of the phthalate restriction.

We visited all the companies before the sample collection campaign began in order to identify the most significant work tasks with respect to phthalate exposure. Altogether, 20 workers aged 24–61 participated in the study (16 men and 4 women). Three men were smokers.

The control population consisted of 60 occupationally non-exposed volunteers (aged 25–63; 42 women and 18 men) from the Helsinki, Kuopio, and Tampere regions. One spot urine sample (first-morning void) was collected from these volunteers [9].

The study design was given to all the participants in written form, and each gave their written consent. The study was approved by the local Coordinating ethics committee (HUS Joint Authority, Helsinki, Finland) (reference 347/13/03/00/149).

### 2.2. Workers’ Urine Sample Collection 

The participants were given instructions to collect 5 spot urine samples as follows. (i) First-morning-void after a 2‒4-day holiday. Since phthalates have a short biological half-life [8,10], we expected that after inhalation exposure, all the phthalate metabolites would be excreted into the urine within two days. Accordingly, this sample represents the individual background level of the participant. The samples collected during day shifts were (ii) pre-shift (first-morning-void), (iii) post-shift (collected immediately after the shift), (iv) post-shift evening (collected around 7–8 pm), and (v) next-morning (first-morning-void). Equivalent sample collection times were set for other work shifts. In most cases, at least one work shift preceded the sample collection day.

The sample collection took place in 2015. The urine collection vessels and storage vessels were made of polypropylene, polystyrene, polyethylene, or glass. The urine samples were stored as frozen until analysis (−20 °C).

In order to take urinary dilution into account, the phthalate metabolite concentrations were normalized to specific gravity and adjusted to creatinine excretion. Normalization was carried out by multiplying the original concentration with correction factor k_d_ = (1.021−1)/[d(sample)−1], where the d(sample) was the specific gravity of the urine sample. The value of 1.021 is the average urine specific gravity value of the Finnish working-age population (based on measurements at FIOH; data not shown). In statistical calculations, we replaced urinary values below LOQ with values of LOQ/2 [11]. Because of the low numbers of workers, no statistical tests were applied. Urinary specific gravity was measured using a digital urine specific gravity refractometer. Urinary creatinine concentrations were measured using a colorimetric method.

### 2.3. Standards and Reagents

The native and isotope-labeled standards for MiNP were acquired from Cambridge Isotope Laboratories, Inc. (Andover, MA, US). The native and isotope-labeled standards for cx-MiOP and cx-MiNP were purchased from Santa Cruz Biotechnology, Inc. (Dallas, TX, US), and for OH-MPHP from Toronto Research Chemicals, Inc. (Toronto, ON, Canada). The mehanol was purchased from J.T. Baker (Deventer, the Netherlands), and the acetic acid was purchased from VWR International Ltd. (Espoo, Finland). The glucuronidase/sulphatase enzyme solution (Helix pomatia H2; glucuronidase activity 304199 units/ml, sulphatase activity 2976 units/ml) was obtained from Sigma-Aldrich (St. Louis, MO, USA). The diisodecyl phthalate was purchased from Chiron AS (Trondheim, Norway), and the diisononyl phthalates from LGC Standard GmbH (Wiesel, Germany) and Chiron AS. The internal standard D4-diisononyl phthalate and di(2-propylheptyl) phthalate were from Sigma Aldrich, and the formic acid up to 98% from Fluka (Buchs, Switzerland). The structures of phthalates and their metabolites are provided in Supplementary Figure S1.

### 2.4. Analysis of Phthalate Metabolites in Urine

Total phthalate metabolite concentration was measured (free + conjugated). For quantitation at the beginning of the analytical procedure, isotope-labeled internal standards for phthalate metabolites (5.0 ng of each) were added to the 0.25 ml urine sample. The sample volume was adjusted to 0.50 ml with water, consisting of 0.05 µL glucuronidase/sulphatase solution (Helix pomatia H2; glucuronidase activity 304,199 units/ml, sulphatase activity 2976 units/ml). The enzyme reaction (deconjugation) took place at 37 °C for 20 h. Next, the hydrolysis sample was centrifuged using Eppendorf 5810 (Hamburg, Germany) at 2600 g for 10 min. Prior to the instrumental analysis, 100 µL of the hydrolyzed sample was transferred to an autosampler vial, and 10 µL of 1% aqueous acetic acid was added. Phthalate metabolites were separated and quantified using the Thermo Scientific UltiMate 3000 Rapid Separation LC system (Germering, Germany) connected to a Thermo Finnigan TSQ Quantum Discovery MAX triple quadrupole mass spectrometer (Waltham, MA, USA). The details of the LC-MS/MS conditions are given in Supplementary Table S1. The limit of quantification (LOQ) for all the studied metabolites was 1.0 μg/L.

### 2.5. Workplace Air Sample Collection

The air samples were collected at the workplaces to measure DiNP, DiDP, and DHPH concentrations during the production processes. Stationary and/or personal breathing zone (PBZ) samples were collected into Tenax TA® OVS tubes (SKC-226-56, SKC Inc.). OVS samplers can collect both particulate and gas-phase compounds. Calibrated pumps were used in sample collection with an airflow of 1 l/min. After collection, the samples were stored in a freezer (−20 °C) until analysis.

### 2.6. Analyses of Phthalates in Air Samples

Before the analysis, the adsorbent materials were transferred to glass test tubes. The upper section—glass fiber filter, Tenax resin, and polyurethane foam (PUF)—were moved to a test tube and the lower section—Tenax resin and PUF—to another tube. Methanol 2 ml with an internal standard (D4-diisononyl-phtalate) was added, and the mixture was extracted in an ultrasonic bath for 15 minutes. After extraction, the mixture was filtered through a 0.45 µm filter (Millex HV PVDF, EMD Millipore) into a glass vial.

The standards were made by spiking a known quantity of the standard solution into the OVS-sampler and drawing air through the sampler for 5 minutes at a flow rate of 100 ml/min. Standards were extracted and treated as samples.

The analysis was performed using a liquid chromatography tandem-mass spectrometry (LC-MS/MS) technique. The instrumentation used was Thermo Finnigan Surveyor liquid chromatography with Thermo Quantum Ultra triple quadrupole mass spectrometry. The analytical conditions were as follows: 100 mm × 2.1 mm, 3.5 µm Waters XTerra C18 column, injection volume of 10 µL, and the mobile phase with a flow rate of 0.3 ml/min. The mobile phase consisted of aqueous 5% methanol with 0.1% formic acid (eluent A) and aqueous 95% methanol with 0.1% formic acid (eluent B), gradient program: 0–30 min, 50% B; 30–42 min, from 50% to 100% B; 42–43 min from 100% to 50% B; 43–50 min 50% B.

The standards and samples were scanned through an m/z area of 100–600. All the monitored phthalates occurred as sodium adducts. The ions selected quantification were m/z 441 for DiNP, m/z 469 for DiDP and DPHP and m/z 445 for D4-DiNP.

### 2.7. Risk Assessment

We compared the measured biomarker levels to the biomonitoring equivalents (BEs) calculated on the basis of published health-based limit values, as described by Angerer and co-workers [12]. The basic formula for the calculation of the BE is:

(1) $$C_{ss}=\frac{D\times BW\times F_{ue}}{V_{24}}$$

where C_ss_ corresponds the urinary level of the substance at steady state, D is the external dose as mg/kg bw (in this case either DNEL or RfD,), BW is the body weight (70 kg), V_24_ is the estimated average 24-hour urinary volume (1.7 L), and F_ue_ is the mass fraction of the metabolite excreted into the urine during 48 hours. For cx-MiOP, a F_ue_ of 8% was used, based on the study by Anderson et al. [8]. A F_ue_ of 8% was also used for OH-MPHP, based on the study by Leng et al. [13]

## 3. Results and Discussion

We studied the occupational phthalate exposure of plastics workers in four different factories. The biomonitoring results of the workers were normalized to a specific gravity in order to take the urinary dilution into account. The profiles of the urinary phthalate metabolite concentration of the workers were also similar when creatinine-adjusted concentrations were used (data not shown).

It should be noted that in the present work, it was not possible to differentiate exposure to DPHP and other phthalates containing ester chains of 10 carbons (like DiDP). Accordingly, the biomonitoring results of monohydroxy propylheptyl phthalate (OH-MPHP, metabolite of DPHP) represent the urinary concentrations of all the hydroxy metabolites of the C10-phthalates.

### 3.1. Cable Factory

The cable factory produced polyvinyl chloride (PVC)-coated cables that contained either DPHP or DiDP as a plasticizer. All five workers worked in the same factory warehouse operating, for example, two extruders. The workers were near the extruders, almost only when the process was about to start and end. The temperature of the closed process was 160–180 °C. Emissions in the air occurred almost entirely only at the points of the extruder line where the cable partly crossed the open cooling line, and local exhaust ventilations (LEVs) were located at these points. The workers wore protective clothing and occasionally also protective gloves (when handling hot machine parts). Respiratory protective equipment (RPE) was not used.

On the sampling day, the DPHP was used as a plasticizer. Table 1 presents the urinary OH-MPHP concentrations of workers split into different sampling times. The urinary OH-MPHP concentrations of the occupationally non-exposed control population (*n* = 60) are provided for comparison [9]. The urinary concentrations of the after-holiday and pre-shift samples (both first morning voids) were mostly below the LOQ. All the urinary concentrations of the non-exposed control population were below the LOQ (1.0 µg/L). The post-shift urinary concentrations of the workers were all above the LOQ, and the maximum was 7.8 µg/L. In addition, in most of the evening and next-morning samples, the urinary OH-MPHP concentration was above the LOQ. The results indicated small-scale occupational exposure.

No occupational DPHP exposure data have been published earlier [1]. Gries, Schütze, and co-workers [14,15] have reported OH-MPHP urinary concentrations below 1 µg/L for the general population. This is in line with the data of our previous study: The urinary concentrations of the occupationally non-exposed population were below 1 µg/L [9]. In addition, pre-shift samples of the plastics workers were, in most cases, below LOQ.

As mentioned above, DiDP was also occasionally used in the cable factory. Thus, whether the urinary monocarboxy isononyl phthalate (cx-MiNP) concentration in the urine of workers differed from that in the urine of the control population was of interest. Ninety-two percent of the urinary cx-MiNP concentrations of non-exposed controls were below LOQ—the maximum concentration was 3.4 µg/L [9]. A detectable amount of cx-MiNP was found in urine of four of the five workers. In the case of three workers, the data range was <LOQ to 3.4 µg/L, which is of the same level as the respective data of the control population. However, the urinary cx-MiNP concentration range of one worker was 1.8 to 14.7 µg/L, and the highest concentration was detected in the after-holiday sample. The second-highest concentration (11.8 µg/L) was that of the pre-shift sample. This might indicate exposure during the previous working day or earlier. However, as discussed above, due to the lack of separation power of the analytical separation method used, we cannot reliably differentiate exposure to DPHP and DiDP. Thus, although we assume that this was mainly derived from exposure to DiDP, we cannot exclude the contribution of DPHP to the cx-MiNP results. The fact that there was limited quantitative information on the proportion of different DiDP metabolites excreted in urine further complicates the assessment. 

We collected two air samples at the cable factory. Because the workers were only occasionally present at the production lines, we decided to collect two static air samples at the cooling points of the lines (located next to the LEVs). Both DPHP and DiDP were analyzed. The results of the first extruder were <0.01 mg/m^3^ (DPHP) and 0.07 mg/m^3^ (DiDP). The respective results of the second extruder were 0.15 mg/m^3^ and 0.02 mg/m^3^. The results showed some emission of DPHP in the second extruder, regardless of the LEV. After conversion, the 8 h time-weighted average (TWA) value was 0.03 mg/m^3^. This was only 2% of our estimated DNEL_workers_ of 1.45 mg/m^3^ (see Supplementary Table S2) and two to three orders of magnitude lower than the DNEL_workers_ given by the REACH registrants of DPHP (5 mg/m^3^ for local irritation and 35.3 mg/m^3^ for systemic effects, [16]). The difference between our DNEL and the DNEL given by REACH registrants was that our DNEL was based on thyroid effects identified as critical effects by Bhat et al. [17], whereas industry had not considered these effects relevant. Interestingly, a small amount of DiDP was detected in both air samples.

### 3.2. Plastics Producing Company

In this company, PVC plastics are manufactured using both DPHP and DiNP as plasticizers (in different products though). Four of the five workers mixed PVC resins and additives, and granulated and packed the products. One worker worked on product development in the laboratory and performed small scale mixing. The production procedures were almost entirely automatized and closed, and the workers were near the machines only when starting and ending the process and when cleaning the machines. The workers used protective clothing and gloves—however, some of them wore short-sleeved shirts. The laboratory worker occasionally used nitrile gloves. RPE was not used.

Table 2 presents the urinary OH-MPHP concentrations of the workers by sampling time. The urinary OH-MPHP concentrations of the control population are provided for comparison. The urinary concentrations of the after-holiday samples (first morning voids) were mostly below the LOQ. All the urinary concentrations of the non-exposed controls were below the LOQ. Most of the post-shift urinary concentrations of the workers were above the LOQ, and the maximum was 21.0 µg/L. All the evening samples contained measurable amounts of OH-MPHP (maximum 25.4 µg/L), and in most of the next-morning samples, the urinary OH-MPHP concentration was still above the LOQ (maximum 8.2 µg/L). Similar to the results of the cable factory, these results indicated small-scale occupational exposure.

All the urinary monoisononyl phthalate (MiNP) results were below LOQ, but monocarboxy isooctyl phthalate (cx-MiOP) concentrations were detected in almost all the urine samples (Table 3). This was not surprising as 98% of the urinary cx-MiOP concentrations of the non-exposed control population were above LOQ [9]. The P95 value of the control data was 32.3 µg/L, which can be considered a reference value (RV) for occupational exposure. The maximum concentrations in the workers’ after-holiday, pre-shift, and post-shift samples were all clearly below the P95 value of the control population. On the other hand, the maximum in the post-shift evening samples (43.1 µg/L) was above the P95 of the controls, which would indicate small-scale occupational exposure. However, the maximum concentration remained below the maximum urinary concentration of the control population (132 µg/L, Table 3). The maximum in the next morning samples was again below the P95 value of the control population.

It should be noted that the DiNP used at the company also contained small amounts of C10-phthalates (like DiDP and DPHP). Although we assume that the OH-MPHP results (Table 2) are mainly derived from exposure to DPHP, we cannot exclude the contribution of DiDP to the OH-MPHP results.

Occupational exposure to DiNP also occurred in a German car factory [3]. The median cx-MiOP concentration of the workers’ post-shift samples (57.8 µg/L) was 11 times higher than the median of the control group (administrative staff from the same factory). The urinary cx-MiOP concentration of the workers was in the range of 24.7 to 286 µg/L, which was clearly higher than the overall concentration range detected in the present work (<LOQ-43.1 µg/L).

Three air samples were collected at the plastics producing company. In this case too, the workers were only occasionally present at the production lines, thus we decided to collect only static air samples. Two samples were collected in the factory and one in the laboratory. One of the factory samples was collected by the granulating machine and the other at the site at which the raw materials feeding took place. The DPHP and DiNP concentrations in all the three air samples were below LOQ 0.01 mg/m^3^. This indicated that the small-scale occupational exposure, especially in the case of DPHP, was mainly from skin contact (including hand-to-mouth exposure).

### 3.3. Producer of Coated Textiles

This company manufactured PVC-coated textiles and used DiNP and DPHP as plasticizers. Only DiNP was used during the sampling date. Eight workers took part in the study—six of them working in the factory, one in the laboratory, and one was a production manager. The factory workers prepared the coating material (two workers), operated an extruder (two workers), and other machines (two workers). Protective clothing but no RPE was used.

The primary metabolite of DiNP (MiNP) was not detected in any of the workers’ urine samples, which was also the case with the control population samples. The secondary metabolite of DiNP (cx-MiOP), on the other hand, was detectable in almost all the urine—only in two post-shift samples was cx-MiOP concentration below the LOQ (Table 4). The median concentrations of all the workers’ urine samples (range 5.9 to 13.1 µg/L) were higher than the median of the control population (4.9 µg/L). The same was true for GMs, except those of the workers’ post-shift samples. Interestingly the highest medians were those of the after-holiday and pre-shift samples (Table 4). Although it is possible that workers carry chemicals into their homes via clothing, resulting in exposure also during the holidays, we consider it more likely that the elevated levels observed after holidays might reflect delayed absorption and excretion due to the slower dermal exposure. This was also observed in the case of bisphenol A [18]. As discussed above, the P95 value of the control population data (32.3 µg/L) can be considered an RV for occupational exposure. The P95 values of the workers’ post-shift, post-shift evening, and next-morning samples were all above the P95 values of the control population. This indicates occupational exposure to DiNP. The P95 value of the pre-shift samples (70.1 µg/L) was also above that in the control populations’ data. This might indicate exposure during the previous working day.

The highest cx-MiOP concentrations were detected in the samples of one extruder operator. The post-shift evening and next-morning samples of one machine operator also contained cx-MiOP concentrations slightly above the P95 level of the control population.

As mentioned above, DPHP was also occasionally used in the factory. The after-holiday samples of two workers contained a small amount of OH-MPHP, but their other samples did not contain this metabolite. A small amount of OH-MPHP was detected in the urine samples of one extruder operator (not detected in the after-holiday sample), and in the post-shift evening and next-morning samples of one machine operator. The maximum OH-MPHP concentration of all the samples was 5.2 µg/L. No detectable amounts of OH-MPHP were found in the urine samples of the control population (Table 1). Accordingly, the low-level OH-MPHP concentrations detected in some of the worker’s urine samples indicated occasional use of DPHP at the factory.

DiNP and DPHP concentrations were also analyzed in the air samples collected at the factory. PBZ samples were collected from five of the six factory workers, and one static sample was collected near the control room of the extruder. All the air concentrations were below LOQ 0.01 mg/m^3^. Once again, this indicated that the small-scale occupational DiNP exposure was mainly from skin contact.

### 3.4. Tarpaulin Producer

This company reprocessed PVC-coated tarpaulins—the tarpaulin material was manufactured elsewhere. The PVC plasticizer was DiNP. Two workers processed tarpaulins (cutting, seaming, perforation, etc.) in a hangar space. Short-sleeved working clothes were used without any PPE.

All the MiNP results were below the LOQ. All the urine samples of the workers contained detectable amounts of cx-MiOP (range 6.9 to 33.2 µg/L). The highest concentration was in one after-holiday sample. The level of DiNP exposure was the same as that of the non-occupationally exposed control population.

One stationary air sample collected at the company contained <0.02 mg/m^3^ of DiNP (i.e., concentration was below the LOQ).

### 3.5. Risk Assessment

ECHA evaluated the scientific data on the toxicity of DiNP in 2013 [19]. Although DiNP has shown anti-androgenic properties, its potency is considerably lower than that of di(2-ethylhexyl) phthalate (DEHP) [20]. DiNP has also caused liver effects in rodents, which ECHA has used as critical effects for setting a DNEL for DiNP. The DNEL given by ECHA for the general population was 0.075 mg/kg for oral and 0.35 mg/m^3^ for inhalation exposure. We have previously calculated a biomonitoring equivalent (BE) of 250 µg/L for cx-MiOP corresponding to daily exposure of 0.075 mg/kg [9]. This was done using the urinary mass-balanced based formula presented in the Materials and Methods. A F_ue_ of 8% was used as an estimate of the proportion of the cx-MiOP excretion in the urine. No authorities have given any health-based limit values for DPHP, but Bhat et al. [17] have proposed a health-based reference value of 0.1 mg/kg bw/day for the general population exposure to DPHP, based on the thyroid effects seen in rodents. We have calculated a biomonitoring equivalent (BE) of 330 µg/L for OH-MPHP corresponding to this reference value [9]. A F_ue_ of 8% was used as an estimate of the proportion of the OH-MPHP excretion in the urine. Neither ECHA nor Bhat et al. [17] derived a DNEL for workers’ inhalation exposure. Supplementary Table S2 shows our estimated inhalation DNELs and corresponding BEs for worker’s inhalation exposure using the same key studies and following ECHA R8 guidance on the characterization of the dose [concentration]-response for human health [21]. The resulting DNELs for workers’ inhalation exposure were 2 mg/m^3^ and 1.45 mg/m^3^ and BEs 900 and 700 µg/L for DiNP (cx-MiOP) and DPHP (OH-MPHP), respectively.

When these were compared to the measured urinary phthalate levels, we could conclude that even the maximum levels remained well below any of the BE values (see Supplementary Table S3). This suggested a negligible health concern for these workers. It should also be noted that these measured levels represented peak excretion rather than steady-state urinary excretion. Since BE levels corresponded to steady-state excretion (or daily average exposure), this approach was likely to result in overestimation of the daily exposure and risk. However, regardless of these inaccuracies, the approach was useful for screening purposes; if the Risk Characterisation Ratios (RCRs, i.e., measured biomarker levels divided by BE values) remain low even when using this approach, there was no need for further refinement of the assessment. 

## 4. Conclusions

This study presents data on the internal exposure to DiNP and DPHP in four companies that use phthalates in their PVC production or reprocessed PVC plastics. In total, 20 workers took part in the research, and five urine samples were collected from each of them: After-holiday, pre-shift, post-shift, evening, and next-morning samples.

In both the cable factory and the plastic producing company, the urinary OH-MPHP concentrations of the after-holiday and pre-shift samples were mostly below the LOQ at 1.0 µg/L. Most of the post-shift, evening, and next-morning urinary concentrations of the workers were above the LOQ (maximum 21 µg/L). Given that all the urinary OH-MPHP concentrations of the non-exposed control population were below the LOQ [9], these results indicate small-scale occupational exposure to DPHP. When taking into account the low or non-detectable levels of DPHP in the air samples, we can conclude that skin contact has likely contributed to the total exposure.

DiNP was also used in the plastic producing company. Urinary cx-MiOP concentrations were detected in almost all the urine samples of the workers, which was in line with the control population data (98% of the urinary cx-MiOP data were above the LOQ [9]). The P95 value of 32.3 µg/L of the control data can be considered an RV for occupational exposure. The maximum concentrations in the workers’ after-holiday, pre-shift, and post-shift samples were all clearly below the RV. On the other hand, in textile manufacturing workers, elevated levels of cx-MiOP were seen in their pre-shift, post-shift, evening, and next-morning samples. The highest levels were observed in the next morning samples, which may be due to delayed absorption via the skin. 

Two workers who processed tarpaulins were not occupationally exposed to DiNP. The air sample collected at the company contained a DiNP concentration below the LOQ.

As is typical with occupational exposure studies, the number of participants in this study was relatively low. However, the results increase our current knowledge on the exposure to these phthalates in plastic product manufacturing and suggest that under these operating conditions, exposures remain low; well below the biomonitoring equivalents estimated on the basis of available health-based limit values. However, further data are needed to obtain a broader overview of occupational exposure to these phthalates in different exposure scenarios.

## Figures and Tables

**Table 1:** Total urinary monohydroxy propylheptyl phthalate (OH-MPHP) concentrations of workers in cable factory (*n* = 5) by sampling time. First-morning-void concentrations of occupationally non-exposed control population (*n* = 60) are provided for comparison [9]. Concentrations (µg/L) were normalized to a specific gravity of 1.021.

	Workers (Cable Factory)					Non-Exposed
	After Holiday	Pre-Shift	Post-Shift	Post-Shift Evening	Next Morning	
AM	^-^	^-^	4.1	4.0	2.6	^-^
GM	^-^	^-^	3.5	2.7	2.1	^-^
GSD	^-^	^-^	1.9	3.0	2.4	^-^
Min	<LOQ	<LOQ	1.8	<LOQ	<LOQ	<LOQ
Median	^-^	^-^	3.9	2.7	2.5	^-^
P95	^-^	^-^	7.2	7.7	4.7	^-^
Max	3.5	0.9 ^a^	7.8	7.9	5.2	<LOQ

AM, arithmetic mean; GM, geometric mean; GSD, geometric standard deviation; P95, 95th percentile; LOQ, limit of quantitation. ^-^ Not calculated because either most of or all results were <LOQ. ^a^ Normalized result was <LOQ, but the original result, without any adjustments, was >LOQ.

**Table 2:** Total urinary OH-MPHP concentrations of workers in plastics producing company (*n* = 5) by sampling time. First-morning-void concentrations of occupationally non-exposed control population (*n* = 60) are provided for comparison [9]. Concentrations (µg/L) were normalized to a specific gravity of 1.021.

	Workers (Plastics Producer)					Non-Exposed
	After Holiday	Pre-Shift	Post-Shift	Post-Shift Evening	Next Morning	
AM	^-^	1.9	8.7	12.7	3.7	^-^
GM	^-^	1.3	4.7	8.3	2.1	^-^
GSD	^-^	2.8	4.4	3.3	3.8	^-^
Min	<LOQ	<LOQ	<LOQ	1.4	<LOQ	<LOQ
Median	^-^	1.0	7.1	11.2	4.5	^-^
P95	^-^	4.2	19.3	24.5	7.6	^-^
Max	2.0	4.4	21.0	25.4	8.3	<LOQ

For abbreviations, see Table 1. ^-^ Not calculated because either most of the results or all results were <LOQ.

**Table 3:** Total urinary monocarboxy isooctyl phthalate (cx-MiOP) concentrations of workers in plastics producing company (*n* = 5) by sampling time. First-morning-void concentrations of occupationally non-exposed control population (*n* = 60) are provided for comparison [9]. Concentrations (µg/L) were normalized to a specific gravity of 1.021.

	Workers (Plastics Producer)					Non-Exposed
	After Holiday	Pre-Shift	Post-Shift	Post-Shift Evening	Next Morning	
AM	10.8	6.0	9.1	16.4	13.9	11.4
GM	8.8	3.6	7.6	12.1	10.9	5.8
GSD	2.2	3.7	2.0	2.3	2.2	2.7
Min	2.4	<LOQ	3.7	5.1	4.2	<LOQ
Median	11.8	4.0	6.4	11.9	13.4	4.9
P95	16.9	12.3	16.3	37.6	26.9	32.3
Max	17.1	12.8	16.8	43.1	29.4	132

For abbreviations, see Table 1.

**Table 4:** Total urinary cx-MiOP concentrations of workers in the company producing coated textiles (*n* = 8) by sampling time. First-morning-void concentrations of occupationally non-exposed control population (*n* = 60) are provided for comparison [9]. Concentrations (µg/L) were normalized to a specific gravity of 1.021.

	Workers (Producer of Coated Textiles)					Non-Exposed
	After Holiday	Pre-Shift	Post-Shift	Post-Shift Evening	Next Morning	
AM	14.4	22.2	13.4	21.4	26.2	11.4
GM	11.6	13.2	5.5	10.3	12.2	5.8
GSD	2.0	2.7	5.3	3.3	3.4	2.7
Min	4.4	4.9	<LOQ	3.5	2.9	<LOQ
Median	13.1	10.4	5.9	7.3	9.9	4.9
P95	31.1	70.1	40.3	74.7	94.9	32.3
Max	37.8	87.9	49.3	94.5	126	132

For abbreviations, see Table 1.

## Supplementary Materials

The following are available online at https://www.mdpi.com/1660-4601/17/6/2035/s1, Figure S1: Structures of the studied phthalates and their metabolites, Table S1: Monitored precursor and product ions, and collision energies (CE) for LC-MS/MS, Table S2: Setting of inhalation DNEL and corresponding biomonitoring equivalent (BE) for workers’ exposure to DiNP and DPHP, Table S3: Biomonitoring equivalents (BEs) and Risk Characterization Ratios (RCRs) for DiNP and DPHP. RCRs have been calculated using maximum levels measured in exposed workers.
