# Supplementary material for: Occupational Exposure of Plastics Workers to Diisononyl Phthalate (DiNP) and Di(2-propylheptyl) Phthalate (DPHP) in Finland
Source: Int J Environ Res Public Health. 2020 Mar 19;17(6):2035. doi: 10.3390/ijerph17062035 (PMC7143504; doi:10.3390/ijerph17062035)
Supplement: Supplementary file 1 [file ijerph-17-02035-s001.pdf]

Supplementary information - International Journal of Environmental Research and Public Health (IJERPH), 2020

Occupational exposure of plastics workers to diisononyl phthalate (DiNP) and di(2-propylheptyl) phthalate (DPHP) in Finland

Simo P. Porras[a,*], Minna Hartonen[a], Jani Koponen[b], Katriina Ylinen[a], Kyösti Louhelainen[a], Jarkko Tornaeus[a], Hannu Kiviranta[b], Tiina Santonen[a]

[a] Finnish Institute of Occupational Health, PO Box 40, FI-00032 Työterveyslaitos, Finland

[b] Finnish Institute for Health and Welfare (THL), PO Box 95, FI-70701 Kuopio, Finland

* Correspondence: simo.porras@ttl.fi

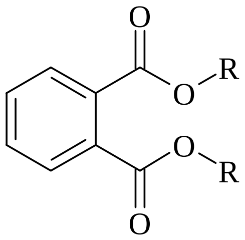

parent phthalate (R)

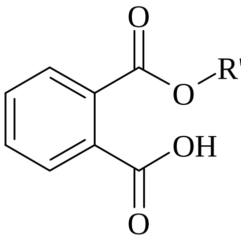

analyzed phthalate metabolite (R’)

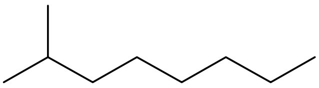<br>diisononyl phthalate (DiNP)	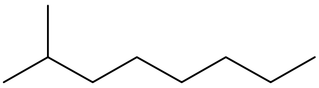<br>monoisononyl phthalate (MiNP)<br>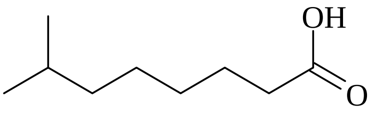<br><br>monocarboxy isooctyl phthalate (cx-MiOP)
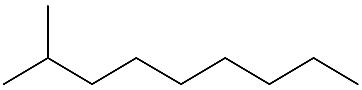<br>diisodecyl phthalate (DiDP)	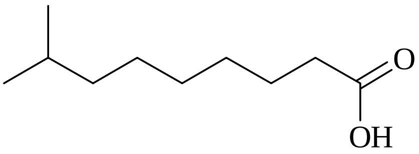<br><br>monocarboxy isononyl phthalate (cx-MiNP)
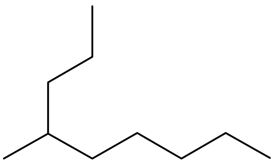<br>di(2-propylheptyl) phthalate (DPHP)	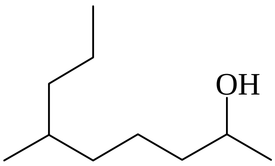<br><br>monohydroxy propylheptyl phthalate (OH-MPHP)

Figure S1. Structures of the studied phthalates and their metabolites.

Materials and methods

LC-MS/MS conditions

We used a 50 mm × 2.1 mm, 3.5 µm Waters XBridge C18 column and mobile phase with a flow rate of 0.25 ml/min. The mobile phase consisted of aqueous 10% methanol (eluent A) and 100% methanol with 0.1 % acetic acid (eluent B), and the gradient programme was as follows: 0.0–1.7 min, 17% B; 1.7–3.7 min, from 17% to 37% B; 3.7–7.5 min, an isocratic 37% B; 7.5–9.5 min, from 37% to 62% B; 9.5–14.5 min, an isocratic 62% B; 14.5 –16.0 min, from 62% to 95% B; followed by an isocratic elution for 4 min and then return to the initial conditions for 5 min before the next injection. The column oven temperature was set at + 33°C. Mass spectrometry was operated in negative ESI mode with a spray voltage of −2.4 kV, capillary and vaporiser temperatures of 330°C and 300°C, respectively, sheath gas pressure of 80 psi and auxiliary gas pressure of 4 arbitrary units (AU). All the chromatograms were recorded in selected reaction monitoring (SRM) with a specific transition per analyte. The specific quantifier precursor and product ions, as well as the collision energies, are shown in Table S1.

Table S1. Monitored precursor and product ions, and collision energies (CE) for LC-MS/MS.

Analyte	12C transitions (m/z)	2H/13C transitions (m/z)	CE (V)
MiNP	291 → 77	295 → 79	18
cx-MiOP	321 → 173	325 → 173	16
cx-MiNP	335 → 187	339 → 187	16
OH-MPHP	321 → 121	325 → 125	16

Results and discussion

Table S2. Setting of inhalation DNEL and corresponding biomonitoring equivalent (BE) for workers' exposure to DiNP and DPHP.

DiNP	
Point of departure (PoD)	PoD used by ECHA [1] as a starting point for the DNEL for general population was NOAEL=15 mg/kg (oral exposure)
Modification of the starting point	Transforming oral NOEAL to inhalation NOEAC (following the approach given in [2]):<br>allometric scaling factor = 4<br>BW=70 kg<br>inhaled volume =10 m^3<br>exposure time is 5 days per week (factor 7/5)
Adjustment for absorption via inhalation	Absorption via oral route is 50%, and via inhalation 75% [1] (factor 50/70)
Assessment factors (AFs) for remaining uncertainties	Default AF of 2.5 is used for toxicodynamic differences and the default AF of 5 is used for interindividual differences.
$DNEL_{worker}$ -inhalation	15 mg/kg x 1/4 x 70 kg/10m^3 x 7/5 x 50/75 x 1/2,5 x 1/5 = 1,96 ~ 2 mg/m^3 (corresponding inhaled dose of 0.28 mg/kg)
BE for cx-MiOP	F_{ue}= 8%, 0.28 mg/kg x 70 kg x 0.08/1.7 l = 0.92 mg/l ~ 900 µg/l
DPHP	
Point of departure (PoD)	Bhat et al. [3] used as a starting point human equivalent $BMDL_{10}$ of 10 mg/kg (includes allometric scaling factor)
Modification of the starting point	Transforming oral NOEAL to inhalation NOEAC (following the approach given in [2]):<br>allometric scaling factor = 1 (already included in $BMDL_{10}$)<br>BW=70 kg<br>inhaled volume =10 m^3<br>exposure time is 5 days per week (factor 7/5)
Adjustment for absorption via inhalation	Absorption via oral route is 50%, and via inhalation 75%. There is no specific data on DPHP, assumed to be the same as in the case of DiNP (factor 50/70)
Assessment factors (AFs) for remaining uncertainties	Default AF of 5 is used for interindividual differences. Bhat et al. [3] used also an additional assessment factor of 3 for extrapolation from subchronic animal data to chronic human exposure and a factor of 3 for incomplete database (total AF 5 x 3 x 3 = 45).
$DNEL_{worker}$ -inhalation	10 mg/kg x 70/10 x 7/5 x 50/75 x 1/45 = 1.45 mg/m^3 (corresponding inhaled dose of 0.21 mg/kg)
BE for OH-MPHP	F_{ue}= 8%, 0.21 mg/kg x 70 kg x 0.08/1.7 l = 0.68 mg/l ~ 700 µg/l

NOAEL=no observed adverse effect level, NOAEC=no observed adverse effect concentration, BMDL=benchmark dose level

Table S3. Biomonitoring equivalents (BEs) and Risk Characterization Ratios (RCRs) for DiNP and DPHP. RCRs have been calculated using maximum levels measured in exposed workers.

Phthalate	Metabolite	$DNEL_{worker}$ (mg/m^3)	BE for workers (mg/l)	Maximum urinary levels (mg/l, from Tables 2 and 4)	RCR
DiNP	cx-MiOP	2	0.9	0.126	0.14
DPHP	OH-MPHP	1.45	0.7	0.0254	0.04

References

1. ECHA *Evaluation of the New Scientific Evidence Concerning DINP and DIDP in Relation to Entry 52 of Annex XVII to Regulation (EC) No 1907/2006 (REACH) - Final review report*; European Chemicals Agency: Helsinki, Finland, 2013. http://echa.europa.eu/documents/10162/31b4067e-de40-4044-93e8-9c9ff1960715 (accessed on 19 March 2020).
2. ECHA *Guidance on information requirements and chemical safety assessment, Chapter R.8: Characterisation of dose [concentration]-response for human health, Version: 2.1*; European Chemicals Agency: Helsinki, Finland, 2012. https://www.echa.europa.eu/documents/10162/13632/information_requirements_r8_en.pdf (accessed on 19 March 2020).
3. Bhat, V. S.; Durham, J. L.; English, J. C., Derivation of an oral reference dose (RfD) for the plasticizer, di-(2-propylheptyl)phthalate (Palatinol (R) 10-P). *Regulat. Toxicol. Pharmacol.* 2014, 70, (1), 65-74. http://doi.org/10.1016/j.yrtph.2014.06.002
